# The cost-effectiveness of implementing in-person and computerized interventions to enhance treatment receipt among drug-involved probationers in two US jurisdictions (Economics of SBI Symposium)

**DOI:** 10.1186/1940-0640-10-S2-O13

**Published:** 2015-09-24

**Authors:** Alexander Cowell, Gary A Zarkin, Brendan Wedehase, Scott T Walters, Faye S Taxman

**Affiliations:** 1 RTI International, RTP, North Carolina, USA; 2 University of North Texas Health Science Center, Fort Worth, Texas, USA; 3 George Mason University, Fairfax, Virginia, USA

## Background

In the United States 1 in 45 adults is under criminal justice supervision in the community, and substance use is prevalent among this population. Treatment for this population has been shown to be generally cost-effective and cost-beneficial. Relatively little is known about the cost-effectiveness of services to enhance treatment receipt among drug-involved probationers. This study assesses the relative cost and cost-effectiveness of face-to-face motivational interview (MI), a motivational computer program (MC), and probation services as usual in a controlled clinical trial. The protocol is to deliver two sessions in the MI and MC arms.

## Material and methods

The analytic perspective is the criminal justice system. To conduct the cost-effectiveness analysis, we calculate incremental cost of obtaining an improvement in effectiveness (incremental cost-effectiveness ratio).

## Results

Preliminary results suggest that using MC to deliver the intervention is cheaper than MI. However, MC may not yield substantial cost savings because the MC cost was higher than anticipated. Reasons may include the considerable effort needed to locate and communicate with probationers to schedule and have them attend a second session as specified in the study protocol. If the MC were delivered in “real world” settings, it is possible that the cost could be considerably lower. Findings on the effectiveness and thus the full cost-effectiveness for the study are pending at the time of submission.

## Conclusions

Probation agencies are faced with both resource constraints and a high proportion of offenders needing substance abuse treatment. Given these constraints, a brief motivational intervention delivered by computer may be appealing on the basis of cost alone. Preliminary findings from this study may temper the appeal of MC when delivered in this format. Effectiveness estimates are needed to determine the degree to which MC is cost-effective.

